# Characteristics of Retracted Research Articles About COVID-19 vs Other Topics

**DOI:** 10.1001/jamanetworkopen.2022.34585

**Published:** 2022-10-04

**Authors:** Xiaoting Shi, Alison Abritis, Rujvee P. Patel, Mikas Grewal, Ivan Oransky, Joseph S. Ross, Joshua D. Wallach

**Affiliations:** 1 Department of Environmental Health Sciences, Yale School of Public Health, New Haven, Connecticut; 2 Retraction Watch, The Center for Scientific Integrity, New York, New York; 3 Yale School of Public Health, New Haven, Connecticut; 4 Yale School of Medicine, New Haven, Connecticut; 5 Arthur Carter Journalism Institute, New York University, New York, New York; 6 Section of General Internal Medicine, Yale School of Medicine, New Haven, Connecticut; 7 Yale-New Haven Hospital Center for Outcomes Research and Evaluation, New Haven, Connecticut; 8 Department of Health Policy and Management, Yale School of Public Health, New Haven, Connecticut; 9 Department of Epidemiology, Rollins School of Public Health, Emory University, Atlanta, Georgia

## Abstract

This cross-sectional study compares the author and journal characteristics of retracted articles on COVID-19 with retracted articles from other topics.

## Introduction

Manuscript retractions represent attempts to officially withdraw published papers or posted preprints that contain errors, fraud, or other kinds of misconduct. Retractions have increased in both number and prominence over the past 2 decades.^1,2^ More recently, concerns have been raised about the number of retracted COVID-19 studies.^3^ While these retractions may be due to greater external scrutiny of COVID-19 literature, little is known about the potential differences between retracted COVID-19 studies and studies on other topics. Accordingly, in this cross-sectional study, we compared author characteristics and reasons for retractions of COVID-19 and non–COVID-19 research articles.

## Methods

We identified all publications and preprints that were marked as retracted, withdrawn, or removed reporting the results of COVID-19 and non–COVID-19–related medical studies that were published or posted since February 1, 2020, and indexed in the Retraction Watch Database (RWDB) as of May 5, 2022.^4^ We excluded nonoriginal research and manuscripts generated by paper mills (ie, companies selling fake manuscripts). For each retracted study, we recorded the publication type and study design, 2020 journal impact factor, first and last authors’ affiliations, date of retraction, and who initiated the retraction. The most prominent reasons for retraction were identified and grouped across similar categories (Table). Three authors (X.S., R.P.P., and M.G.) searched Scopus and then Google Scholar to identify author profiles, which were verified online by institutional affiliation, and recorded each author’s H-index and year of first publication. Fisher Exact and Mann-Whitney *U* tests were used to compare COVID-19 and non-COVID-19 studies (using R version 4.2.0 [R Project for Statistical Computing]). This study followed the Strengthening the Reporting of Observational Studies in Epidemiology (STROBE) reporting guideline and did not require institutional review board approval or informed consent because it did not rely on patient-level data, in accordance with 45 CFR §46.

**Table.** Comparison Between COVID-19 and Non-COVID-19–Related Retractions

Characteristics	Studies, No. (%)			*P* value
	COVID-19 retracted studies (n = 138)	Non–COVID-19 retracted studies (n = 380)	Total (n = 518)	
**Manuscript characteristics**				
Publication type				
Published article	101 (73.2)	380 (100)	481 (92.9)	<.001
Preprint	37 (26.8)^a^	0	37 (7.1)	
Journal impact factor^b^				
Median (IQR)	3.070 (2.113-5.143)	3.466 (2.474-4.703)	3.360 (2.360-4.840)	.36
Without a 2020 impact factor	22 (21.8)	90 (23.7)	112 (23.3)	.79
Study design				
Trial	6 (4.3)	45 (11.8)	51 (9.8)	<.001
Observational study	78 (56.5)	159 (41.8)	237 (45.8)	
Laboratory or preclinical	7 (5.1)	88 (23.2)	95 (18.3)	
Modeling	18 (13.0)	7 (1.8)	25 (4.8)	
Systematic review or meta-analysis	4 (2.9)	25 (6.6)	29 (5.6)	
Other^c^	21 (15.2)	51 (13.4)	72 (13.9)	
Unclear	4 (2.9)	5 (1.3)	9 (1.7)	
Reason for retraction				
Lack of adherence to journal polices or ethics violations	19 (13.8)	69 (18.2)	88 (17.0)	<.001
Duplication of data, image, test, or article	10 (7.2)	44 (11.6)	54 (10.4)	
Errors	20 (14.5)	103 (27.1)	123 (23.7)	
Falsification or fabrication of data, images, or results	0 (0.0)	9 (2.4)	9 (1.7)	
Plagiarism of data, images, text, or article	5 (3.6)	28 (7.4)	33 (6.4)	
Non–misconduct-related other concerns or unspecified	84 (60.9)	127 (33.4)	211 (40.7)	
Retraction requesters				
Authors	47 (34.1)	105 (27.6)	152 (29.3)	<.001
Editors, journals, or publishers	28 (20.3)	158 (41.6)	186 (35.9)	
Other^d^	3 (2.2)	12 (3.2)	15 (2.9)	
Multiple	4 (2.9)	3 (0.8)	7 (1.4)	
Unclear	56 (40.6)	102 (26.8)	158 (30.5)	
Time from publication to retraction, mo				
<6	113 (81.9)	227 (59.7)	340 (65.6)	<.001
≥6	25 (18.1)	153 (40.3)	178 (34.4)	
**Author characteristics**				
Median (IQR) total authors	5 (3-9)	5 (3-7)	5 (3-8)	.55
First author				
Location				
Asia	56 (40.6)	237 (62.4)	293 (56.6)	<.001
Europe	38 (27.5)	70 (18.4)	108 (20.8)	
North America	39 (28.3)	45 (11.8)	84 (16.2)	
Africa	3 (2.2)	15 (3.9)	18 (3.5)	
South America	1 (0.7)	8 (2.1)	9 (1.7)	
Australia	1 (0.7)	5 (1.3)	6 (1.2)	
Affiliation				
Academia or hospital	135 (97.8)	371 (97.6)	506 (97.7)	.40
Government	3 (2.2)	3 (0.8)	6 (1.2)	
Nongovernmental or nonprofit	0	3 (0.8)	3 (0.6)	
Industry	0	3 (0.8)	3 (0.6)	
Other	0	0	0	
Training				
MD	73 (52.9)	157 (41.3)	230 (44.4)	<.001
PhD only	40 (29.0)	49 (12.9)	89 (17.2)	
Other degree only	7 (5.1)	29 (7.6)	36 (6.9)	
No graduate degree or current student	5 (3.6)	19 (5.0)	24 (4.6)	
Unknown/unavailable	13 (9.4)	126 (33.2)	139 (26.8)	
H-index				
Median (IQR)	7 (2-14)	2 (1-5)	3 (1-7)	<.001
No information	7 (5.1)	30 (7.9)	37 (7.1)	.34
Time since first publication				
Median (IQR), y	9 (3-16)	4 (2-9)	5 (2-11)	<.001
No information	7 (5.1)	30 (7.9)	37 (7.1)	.34
Last author				
Location				
Asia	57 (41.3)	238 (62.6)	295 (56.9)	<.001
Europe	35 (25.4)	68 (17.9)	103 (19.9)	
North America	41 (29.7)	49 (12.9)	90 (17.4)	
Africa	3 (2.2)	16 (4.2)	19 (3.7)	
South America	1 (0.7)	7 (1.8)	8 (1.5)	
Australia	1 (0.7)	2 (0.5)	3 (0.6)	
Affiliation				
Academia or hospital	134 (97.1)	370 (97.4)	504 (97.3)	.93
Government	1 (0.7)	2 (0.5)	3 (0.6)	
Nongovernmental or nonprofit	2 (1.4)	3 (0.8)	5 (1.0)	
Industry	1 (0.7)	3 (0.8)	4 (0.8)	
Other	0 (0.0)	2 (0.5)	2 (0.4)	
Training				
MD	76 (55.1)	172 (45.3)	248 (47.9)	.002
PhD only	39 (28.3)	76 (20.0)	115 (22.2)	
Other degree only	6 (4.3)	33 (8.7)	39 (7.5)	
No graduate degree or current student	0 (0.0)	5 (1.3)	5 (1.0)	
Unknown/unavailable	17 (12.3)	94 (24.7)	111 (21.4)	
H-index				
Median (IQR) index score	17 (4-26)	5(1-17)	7 (2-21)	<.001
No information	5 (3.6)	13 (3.4)	18 (3.5)	>.99
Time since first publication				
Median (IQR), y	16 (9-25)	10 (2-19)	13 (3-21)	<.001
No information	5 (3.6)	13 (3.4)	18 (3.5)	>.99

^a^ By preprint repository, these include 23 manuscripts in *medRxiv*, 9 manuscripts in *Social Science Research Network*, 4 manuscripts in *bioRxiv*, and 1 manuscript in* Research Square*.

^b^ Preprints included in analysis did not have an associated impact factor, meaning the denominator for journal impact factor characteristics is 101 for COVID-19 retracted studies and 481 for total studies.

^c^ Other includes case report or case series, and nonsystematic review articles.

^d^ Other includes external readers, institutional review board, and data set owners.

## Results

As of May 5, 2022, 138 COVID-19 and 380 non–COVID-19 retracted studies meeting our inclusion criteria were indexed in the RWDB (Table); 101 COVID-19 studies (73.2%) were published articles (37 [26.8%] were preprints), whereas all 380 non–COVID-19 studies were published articles. COVID-19 studies were more likely to be retracted or withdrawn within 6 months of publication or posting than non–COVID-19 studies (113 of 138 studies [81.9%] vs 227 of 380 studies [59.7%]; *P* < .001). There were also significant differences in study design and reasons for retraction between COVID-19 and non–COVID-19 retracted studies, with the former having a higher proportion of modeling studies and being retracted without detailed explanations or for non–misconduct-related concerns. A greater proportion of retractions of non–COVID-19 studies (158 of 380 [41.6%]) were initiated by editors, journals, or publishers than COVID-19 studies (28 of 138 [20.3%]).

First and last authors of retracted COVID-19 studies had a greater number years since their first publication than those of non–COVID-19 studies (median [IQR] time since first publication: first author, 9 [3-16] years vs 4 [2-9] years; *P* < .001; last author, 16 [9-25] years vs 10 [2-19] years; *P* < .001), as well as higher median H-indices than those of non–COVID-19 studies (first author, 7 [2-14] vs 2 [1-5]; *P* < .001; last author, 17 [4-26] vs 5 [1-17]; *P* < .001).

## Discussion

This cross-sectional study of all retractions of publications and withdrawals of preprints indexed in the RWDB between February 2020 and early May 2022 suggests that there are differences in author characteristics and reasons for retractions between COVID-19 and non–COVID-19 research articles. In particular, COVID-19 retracted studies had more experienced first and last authors. However, COVID-19 studies were more often than not retracted for non–misconduct-related concerns, similar to previous evaluations,^5,6^ which may suggest that COVID-19 studies are subject to a greater level of external scrutiny than non–COVID-19 studies.

This study has limitations. While RWDB is the most comprehensive source of retractions to date, additional retractions and withdrawals may take place or may not yet have been entered into the RWDB by the date of our study. Retraction notices are also not uniform across publishers, which can make it difficult to identify the most prominent reason for each retraction. Although our findings suggest that retracted COVID-19 studies may not be more likely to involve fraud or other kinds of misconduct than non–COVID-19 studies, the large number of studies retracted without detailed explanations emphasizes the need for more uniform retraction notices across publishers.
